# Stepwise ABC system for classification of any type of genetic variant

**DOI:** 10.1038/s41431-021-00903-z

**Published:** 2021-05-13

**Authors:** Gunnar Houge, Andreas Laner, Sebahattin Cirak, Nicole de Leeuw, Hans Scheffer, Johan T. den Dunnen

**Affiliations:** 1 Department of Medical Genetics, Haukeland University Hospital, Bergen, Norway; 2 Medizinisch Genetisches Zentrum, Munich, Germany; 3 Department of Pediatrics, Faculty of Medicine and University Hospital Cologne, University of Cologne, Cologne, Germany; 4 Department of Human Genetics, Radboud University Medical Center, Donders Institute for Brain, Cognition and Behavior, Radboud University, Nijmegen, The Netherlands; 5 Department of Human Genetics and Department of Clinical Genetics, Leiden University Medical Center, Leiden, The Netherlands

**Keywords:** Genetic testing, Diagnostic markers, Genetic testing

## Abstract

The American College of Medical Genetics and Genomics *a*nd the Association for Molecular Pathology (ACMG-AMP) system for variant classification is score based with five classes: benign, likely benign, variant of unknown significance (VUS), likely pathogenic, and pathogenic. Here, we present a variant classification model that can be an add-on or alternative to ACMG classification: A stepwise system that can classify any type of genetic variant (e.g., hypomorphic alleles, imprinted alleles, copy number variants, runs of homozygosity, enhancer variants, and variants related to traits). We call it the ABC system because classification is first functional (A), then clinical (B), and optionally a standard comment that fits the clinical question is selected (C). Both steps A and B have 1–5 grading when knowledge is sufficient, if not, class “zero” is assigned. Functional grading (A) only concerns biological consequences with the stages normal function (1), likely normal function (2), hypothetical functional effect (3), likely functional effect (4), and proven functional effect (5). Clinical grading (B) is genotype–phenotype focused with the stages “right type of gene” (1), risk factor (2), and pathogenic (3–5, depending on penetrance). Both grades are listed for each variant and combined to generate a joint class ranging from A to F. Importantly, the A–F classes are linked to standard comments, reflecting laboratory or national policy. In step A, the VUS class is split into class 0 (true unknown) and class 3 (hypothetical functional effect based on molecular predictions or de novo occurrence), providing a rationale for variant-of-interest reporting when the clinical picture could fit the finding. The system gives clinicians a better guide to variant significance.

## Introduction

Broad genomic testing, using massive parallel sequencing and high-resolution arrays, is the fundamental of next-generation clinical genetics and precision medicine. This development has unleashed an urgent need to share and evaluate genetic variants. The uniqueness of genetic variation has not diminished despite rapidly expanding population-based variant databases, like the Genome Aggregation Database (gnomAD; gnomad.broadinstitute.org). Rare variants are still very common. Of 60,706 individuals in the ExAC Database (http://exac.broadinstitute.org/), 99% of the variants had a frequency of <1% and 54% of the variants were only seen once. For clinical purposes, sharing disease-associated variants is equally important, e.g., in databases like ClinVar (www.ncbi.nlm.nih.gov/clinvar), Global Variome shared LOVD (databases.lovd.nl/shared), and DECIPHER (decipher.sanger.ac.uk). All types of genetic variants should be evaluated in a reproducible and transparent way that makes inter-laboratory interpretations of the same variant as informative as possible. This can be challenging and discrepancies may occur [1–3]. However, a comparison of large US laboratories that used the American College of Medical Genetics and Genomics (ACMG) system showed that concordance in general is good (~90%) [4], especially for high-penetrant variants [5]. Gene- or disease-specific classification systems, which are designed to help, may, in fact, further increase the classification complexity [6–12].

The prevailing system for variant classification was designed by the ACMG and the Association for Molecular Pathology (AMP) [13–15]. While the system works well for high-penetrant dominant variants, it is less well suited for low-penetrant and recessive disease-associated variants, or if the associated phenotypes only partially overlap with the originally reported disease phenotypes. Furthermore, it was not designed for copy number variation [5, 16]. However, systems for CNV classification resulting in ACMG classes have recently been suggested [17, 18].

Since the ACMG system merges functional (molecular) and clinical data into a one-dimensional system, it is not always apparent upon what criteria a classification has been based, unless a classification category list is also provided [1]. In particular, this is problematic for the large ACMG variant-of-unknown-significance (or VUS) group [19, 20], often called a class 3 variant, despite not being on a numerical scale from benign to pathogenic (ACMG does not recommend the use of numbers but letters). In many cases, an ACMG-VUS is of unknown significance due to insufficient evidence to classify it, i.e., lack of relevant information, and it should therefore be treated as such. In some cases, the ACMG-VUS could have a functional effect based on existing data and be in a matching gene for the patient’s phenotype, but still not fulfilling the ACMG criteria for a likely pathogenic variant. Then, a VUS classification may risk delaying or preventing appropriate clinical follow-up that could have clarified the variant’s importance and been of help to the patient and family [20].

Even among experts, the VUS category is used in different ways, and a Dutch study recently reported that a clearly pathogenic variant tended to be labeled a VUS when penetrance was low [21]. Similarly, when one is unfamiliar with medical genetics a variant classified as VUS may easily lead to confusion. As a clinician once remarked: “I think it is called a VUS because the pathogenic mechanism is unknown,” or genetic testing is given up with comments such as “genetics did not give any result.” It is important that a genetic classification is understood the same way among experts (clinical geneticists and clinical laboratory geneticists), other medical specialists, and primary care physicians. That is often not the case today.

Because of the potential risk for misunderstanding, misdiagnosis, and maltreatment, it is an ongoing debate whether a VUS should be reported or not. Opinions are increasingly in line with skeptics saying that VUS reporting may cause more harm than benefit to the patient and family [22]. However, others think that VUS reporting is fine since legal responsibility to follow-up is then shifted from the laboratory to the clinician. International guidelines are needed to structure the decision-making process as to when a genetic variant should be reported or not.

The ABC system for variant classification proposed here makes classification easier because the functional consequences are considered independently from the clinical importance like genotype–phenotype issues. Both stages (A and B) of the grading systems contain a zero when lack of information prevents classification. If desired, ACMG-based classification can be done first, and if the result is a VUS, further evaluation can be done in this system (i.e., is it a VUS grade 0 or 3 in the ABC system, and if grade 3 clinical classification will follow). The system supports the classification of all types of genetic variants including hypomorphic alleles, copy number changes, extensive homozygosity, and regulatory changes, regardless if the variant has a clinical consequence or not. This makes the system universally applicable and versatile, also allowing gene-specific rules from Variation Curation Expert Panels to be applied to the functional dimension when appropriate. The proposed system is still a model, and further testing is planned to determine the robustness and reproducibility.

### Design

**Fig. 1:**
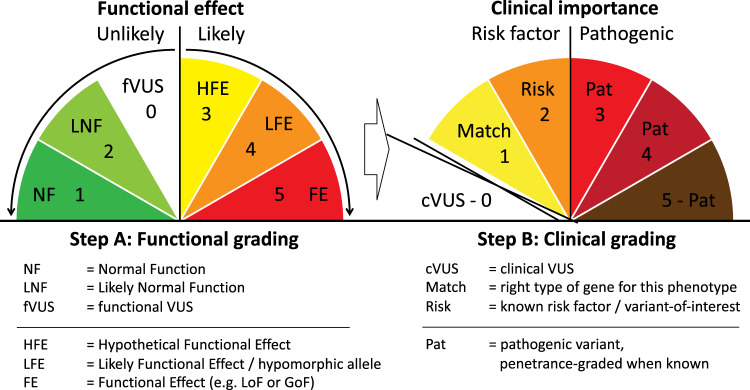
Graphical illustration of step A and B grading. The two scoring steps are functional (**a**) and clinical (**b**). Step A can be viewed as a likelihood scale from normal function to abnormal or no function, the balance being between fVUS and HFE. Step B grades from unknown clinical relevance (cVUS) to pathogenic with high penetrance, “risk” comprising both know risk factor genes and variants of interest (VOI).

In the proposed ABC system, variants are first classified based on known or likely consequences for gene or protein function (step A: functional grading) and subsequently based on known or suspected clinical consequences/correlations (step B: clinical grading) (Fig. 1 and Fig. S1). Functional grading goes from too little information to allow classification (grade 0), to normal function (grade 1), to known functional consequences (grade 5), and concerns both the gene/protein itself as well as expression level (dosage) and place/time of expression (tissue and developmental stage) (Table 1). This functional classification can, if desired, build on the ACMG system or the Sherloc (semiquantitative, hierarchical evidence-based rules for locus interpretation) extension of this system [23], since these systems are compatible with first-dimension classification. Only ACMG criterium PP4 (patient’s phenotype or family history is highly specific for a disease with a single genetic etiology) is not part of the functional grading. Co-segregation or not (ACMG criteria PP1 and BS4) can also be considered in step A grading when such data exist.

**Table 1:** Step A: functional grading.

	Functional classes	Score	~Odds	Description
fVUS	Variant of unknown functional significance—*a functional VUS or fVUS*	0		Variant of unknown *functional* significance—usually due to the lack of knowledge
NF	Normal function (NF)	1	<1%	High-frequency variant with no reason to suspect a recessive or hypomorphic role
LNF	Likely normal function (LNF)	2	1–10%	Moderate-frequency variant with no reason to suspect a recessive or hypomorphic role
HFE	Hypothetical functional effect (HFE)	3	50–90%	Rare variant that could affect gene function based on biological knowledge and bioinformatic tools
LFE	Likely functional effect (LFE), or *Hypomorphic variant* (in recessive disease)	4	90–99%	*Recessive*: variant that reduces gene function, but that only causes a biochemical abnormality or disease if *in trans* to a LoF allele *Dominant*: likely LoF, or variant of likely functional importance
FE	Functional effect (FE)	5	>99%	Variant that disrupt gene function (certain LoF) or known to be disease causing (known GoF or dominant-negative effect)

The Sherloc system is made to merge functional and clinical criteria, but the data they label clinical (e.g., population frequency, de novo occurrence, other explanations for the phenotype, co-occurrence in affected/unaffected) are mostly handled during step A grading in the ABC system. In contrast, step B clinical grading is different and unique to the ABC system (Table 2). It is genotype–phenotype based and goes from too little information to allow clinical grading (grade 0: an unknown, can be applied to any type of gene), to “can be the right type of gene for this phenotype” (grade 1: a potential clinical match), to known risk factor (grade 2: a known low-penetrant variant; a dominant variant with good clinical data in support of a pathogenic role; or a single pathogenic variant in a recessive gene that matches the phenotype), and finally to pathogenic (grade 3). If known, penetrance grading can be specified in the pathogenic group with grade 4 for moderately penetrant variants and grade 5 for highly penetrant variants. Terms like “risk factor,” “variant-of-interest” (VOI), and “pathogenic” are meaningful from a clinical perspective.

**Table 2:** Step B: clinical grading.

	Clinical classes	Score	Description
cVUS	Variant of unknown clinical significance—*a clinical VUS*	**0**	Variant of unknown *clinical* significance, e.g., a variant in a gene that is unlikely to be linked to the patient’s phenotype.
Right type of gene	Variant of potential interest (VOI)	**1**	“The right type of gene” *because the gene fits the phenotype*: (1) dominant variant that could be pathogenic (2) single hypomorphic variant that could be linked to a recessive cause
Risk factor	Known or assumed risk factor variant	**2**	(1) Low penetrance dominant variant (2) Dominant variant with good clinical support of a pathogenic role (3) Single pathogenic variant in a recessive gene that fits the phenotype
Pathogenic	Pathogenic variant	**3**	Pathogenic variant
Pathogenic	Moderate penetrance pathogenic variant	**4**	Dominant pathogenic variant of moderate (20–40%) penetrance
Pathogenic	High penetrance pathogenic variant	**5**	Dominant pathogenic variant of high (>40%) penetrance

Of note, the population frequency of a variant is considered during both A and B grading but from different perspectives. In step A, high variant frequency (the cut-off point for “high” depends on the gene/condition) usually suggests biological tolerance or neutrality, i.e., that there is no counter-selection against this variant in nature. In step B, variant frequency must be compatible with the assumed prevalence of the disease or condition in the relevant population, taking Hardy–Weinberg equilibria and penetrance into consideration. A prerequisite for using Hardy–Weinberg in this setting is a rough idea about disease frequency in the group the individual with the variant belongs to.

There are three major differences between the ABC and the ACMG classification systems. First, the step A functional grading is primarily based on the variant’s effect on gene function or gene expression, not taking clinical consequences into account (Table 1). This makes grading easier, e.g., an enhancer dysfunction causing blue eyes due to tissue-specific low *OCA2* expression can be classified, and the same holds for copy number variants (CNVs), hypomorphic alleles, extensive homozygosity of the autosomal genome (>100 Mb of runs of homozygosity, ROH), imprinted alleles, and common risk factors (like the *F5* “Leiden mutation” c.1601G > A, p.Arg534Gln). Second, the ACMG-VUS class has been split depending on whether there is a lack of information to classify it, i.e., a functional VUS or fVUS with score zero, or whether the available evidence is not sufficient to classify it towards likely benign or likely functional effect, i.e., a variant of hypothetical functional effect (HFE) = a functional grade 3 (Fig. 1 and Table 1). This should help to avoid possible misunderstandings. Third, clinical grading is done of all variants with a functional scoring grade from 3 to 5. Functional grade 0–2 variants are not clinically graded to save time and resources, and because the clinical value of doing this is generally low. However, all these VUS-0 variants should be registered in variant databases.

In step B, clinical grading, genotype–phenotype is considered as well as penetrance. If the gene does not fit the phenotype, the clinical score is zero (0 = a clinical VUS or cVUS). If the gene could fit the phenotype but is not a known disease gene (e.g., not an OMIM morbid gene), or if the phenotype is an uncertain but not unlikely match to a known disease gene, the clinical score is 1 (1 = “right type of gene”). If the variant is a functional grade 3 (HFE, a “VUS+”) and the gene is a known disease gene that could match the phenotype, the clinical grade is 2 (risk factor). In this case, the term “risk factor” just means that the variant is a good phenotype match—there are no assumptions about penetrance involved. In contrast, if the variant is known to have low penetrance (like the *F5* “Leiden mutation”), the clinical grade is also 2 (risk factor). If the variant is definitely pathogenic it is at least clinical grade 3. If the penetrance of a dominant variant is known to be moderate (20–40%), the clinical grade is 4, and if the penetrance of a dominant variant is known to be high (>40%), the clinical grade is 5. If the penetrance is unknown the clinical grade remains at 3. Detailed examples of classification of different types of variants can be found in Suppl. Table S1, an Excel sheet available for download to test the classification of your own variants.

Loss-of-function (LoF) variants can be a common finding in genes that are not dosage-sensitive, often with a gnomAD pLI score of 0 (this score, from 0 to 1, gives a probability for biological intolerance to LoF). A LoF variant in a gene with a pLI of 0 will also be a class 5 variant (known functional effect) because the allele is highly unlikely to produce a functional protein, irrespective of what the clinical consequences are. Such class 5 variants can be of any type (including missense) as long as the functional effect is known. In a recessive setting, the finding may be clinically relevant, while in other settings, it may represent a cVUS, i.e., a random finding just showing carrier status. How variants are placed after functional and clinical grading can be seen in Fig. 1, and more detailed explanations can be found in Tables 1 and 2.

The main challenge of the proposed system is to avoid that relevant variants are overlooked and not adequately appreciated by clinicians. For example, most population-frequent variants will end up in functional categories 1 or 2 (normal/likely normal function), and they do not need further clinical classification. However, exceptions are hypomorphic alleles where carrier frequencies can be high, sometimes with minor allele frequencies (MAFs) in the 0.01–0.05 range. Hypomorphic alleles usually give no phenotype in the homozygous state, but combined with an LoF variant or a deletion a recessive disease may result (e.g., in thrombocytopenia-absent radius syndrome, Stargardt disease, and spondylocostal dysostosis type 5). To avoid being overlooked, step A functional grade of such variants should be 4 (having a likely functional effect) (Table 1). Such hypomorphic alleles must, in general, be known already to be correctly classified—otherwise, they are mostly overlooked until functional or epidemiological studies reveal their significance. Variants that cannot be shifted into grade 1 or 2 because population frequencies are too low, or to grade 3 based on bioinformatic analysis, should be graded 0 (an fVUS). De novo missense variants, not common in databases like gnomAD, should be step A graded as 3 (or higher) to signify that they are of potential interest.

After the functional and clinical grading have been completed (steps A and B), maybe even by the same person if both the molecular and phenotypic knowledge is sufficient, the functional and clinical gradings are combined, forming the basis for a joint variant class from A to F (Table 3) that can be linked to standard variant comments (Table 4). The functional and clinical basis for A to F grading should be stated (e.g., grades 3 + 1 corresponds to class E, see Table 3). A to F grading is common in academia, but in this case, it reflects the clinical significance of a variant finding.

**Table 3:** Combined class based on functional (A) and clinical (B) grading.

A + B class	Grading combinations	Group	Examples of reporting recommendations (policy dependent)
0	Functional grade 0–2	0–2	Not reported—clinical grading unnecessary
F	Functional 3 + clinical 0	3	Not reported if the gene in question is unlikely to be related to the phenotype
E	3 + 1/3 + 2/4 + 0/4 + 1/5 + 0	4–5	*Variant-of-interest (VOI) group*: reporting optional: single variant of potential interest in a gene that could be related to the phenotype (dominant or recessive)
D	3 + 3/4 + 2/4 + 3/5 + 1/5 + 2	6–7	*Low penetrance and good candidate group*: reporting usually recommended
C	4 + 4/5 + 3	8	*Pathogenic*: disease-associated variant, always reported
B	4 + 5/5 + 4	9	*Pathogenic*: disease-associated variant of moderate penetrance
A	5 + 5	10	Pathogenic: disease-associated variant of high penetrance
X	Functional 3–5 + clinical 2–5		Secondary finding/incidental finding/opportunistic finding

**Table 4:** Step C: selection of standard variant comment based on combined class.

Class	Examples of standard comments
0	Normal findings
F	Normal findings—no pathogenic or likely pathogenic variants detected
F/E	Normal findings—no pathogenic variants that could be related to the phenotype were detected
E/D	Normal findings—no pathogenic variants that could explain the phenotype were detected
E/D	Genetic variant of potential interest detected
E/D	Heterozygosity for a recessive genetic variant of potential interest detected
D	A genetic variant that increases susceptibility for this phenotype was detected
C/B/A	Disease-associated pathogenic variant detected (+/− penetrance, if known)
X	Genetic variant unrelated to the clinical question detected

The system includes a class X for secondary/incidental/unsolicited/opportunistic findings and a class 0 when clinical grading has not been performed (because it was not indicated) (Table 3). Note that the choice of comment (from Table 4) depends on the clinical question, i.e., classes D and E can be linked to several (four) standard comments (examples are found in Suppl. Table S1).

The clinical grading can be supported by DECIPHER’s “clinical fit calculator” that takes parameters like genetic heterogeneity, age of disease onset, clinical fit, severity and disease progression, and relevant family history into account (for details, see decipher.sanger.ac.uk). There are also other tools available at the DECIPHER website that can be helpful for variant classification.

## Results

The results after scoring different groups of variants using the ACMG and ABC systems are found in Table 5. Of note, this scoring, as well as the classification in Suppl. Table S1, has been done by the same person (the first author). Accordingly, these tables do not tell anything about classification robustness and reproducibility. Follow-up between-lab comparison studies are planned to investigate this important aspect of any variant classification system.

**Table 5:** Comparison of ACMG and ABC classification in dominant (cases 1–6), recessive (cases 7–11), and SNP-array (cases 12–16) settings.

Case#	Gene/CNV	Finding	Clinical information	ACMG class calculation^a^	ABC	ACMG class vs ABC class
1	*F5*	NM_000130.4:c.1601G > A p.Arg534Gln MAF 0.04–0.07 (Europeans)	A: unexpected deep venous thrombosis at age 56	VUS (PS3, PS4, PP1, BA1)	D/5 + 2	VUS D—susceptibility variant
2	*JAK2*	NM_004972.3:c.2048_2050del p.(Arg683_Glu684insLys) Not in gnomAD v2.1.1	A: high platelet counts, dominant trait in healthy asymptomatic family members	LP (PS4, PP1-M, PM2, PP4)	E/4 + 1	LP E—potential interest variant
3	*UMOD*	NM_001278614.1:c.915C > A p.(Tyr305^a^) Not in gnomAD v2.1.1	B: incidental finding in neonate with cyanosis, unknown if variant is inherited	VUS (PM2, PP3)	E/5 + 0	VUS E—no explanation found
4	*BICD2*	NM_015250.3:c.793A > G p.(Met265Val) 8 in gnomAD v2.1.1	A: family with dominant drop-foot tendency, areflexia and thenar atrophy	VUS (PP1-M, PP3, BS1)	E/3 + 1	VUS E—potential interest variant
5	*BRCA2*	LRG_293t1:c.8177A > G p.(Tyr2726Cys) Not in gnomAD v.2.1.1	A: siblings with ca. ovarii, decreased repair function found in one study	LP (PS4, PM2, PP3, BP1)	D/4 + 2	LP D—susceptibility variant
6	*CACNA1C*	NM_000719.6:c.5852C > G p.(Pro1951Arg) de novo Not in gnomAD v.2.1.1	A: ID with autism, hypospadias and dysmorphic facial features	LP (PS2, PM2, PP2)	E/3 + 2	LP E—no explanation found
7	*FECH*	Chr18(GRCh38):g.5757571588A > G NM_000140.3:c.315-48T > C r.314_315ins315-49_315-1 MAF 0.11 in gnomAD v.2.1.1	A: erythropoietic protoporphyria (EPP), only one variant found	VUS (PS3, PS4, PP1-S, PM3, PP4, BA1)	D/4 + 2	VUS D—potential interest variant
8	*C3*	LRG_27t1:c.4893G > A p.(Trp1631^a^) from father Not in gnomAD v.2.1.1	A: atypical hemolytic uremic syndrome. Only case in family, both parents no signs of aHUS. Five healthy relatives carry the paternal variant in the last exon of C3, and three healthy relatives carry the maternal missense variant.	LP (PS3, PM2, PP3, BS2)	D/4 + 2	LP D—susceptibility variant
	*C3*	LRG_27t1:c.2203C > T p.(Arg735Trp) from mother Not in gnomAD v.2.1.1		VUS (PM2, PP3, BS2)	E/3 + 2	VUS E—potential interest variant
	*C3*	LRG_27t1:c.3346G > A p.(Gly1116Arg) from mother MAF 0.002 in gnomAD v.2.1.1		LB (BP4, BS1)	F/2 + 0	LB F—not further classified
9	*FLVCR2*	NM_017791.2:c.615G > T p.(Trp205Cys) homozygous 1 in gnomAD v.2.1.1	A: aborted fetus with hydranencephaly and cerebral vasculopathy (Fowler syndrome)	VUS (PM2, PP3, PP4)	C/3 + 5	VUS C—pathogenic
10	*CFTR*	NM_000492.3:c.1521_1523del p.(Phe508del) heterozygous	B: aortic aneurysm in young adult	P (PS1, PS3, BS2?)	E/5 + 0	P (incidental finding) E—no explanation found
11	*CFTR*	NM_000492.3:c.1521_1523del p.(Phe508del) heterozygous	A: nasal polyps in young adult	P (PS1, PS3, PS4)	D/5 + 2	PD—susceptibility variant
12	*Dup GRIA3*	NC_000023.10:g.(123183157 _?)_(?_123704413)dup, unknown if dup on X is de novo	A: boy with learning problems and epilepsy	VUS, score 0.30?	E/3 + 1	VUS E—potential interest CNV
13	*Del 15q13.3*	Classic deletion (from BP4 to BP5)	Learning problems, psychosis	P, score 1.00	B/5 + 4	P B—pathogenic
14	*Dup 15q13.3*	Classic duplication (from BP4 to BP5)	Learning problems	VUS, score <0.45	D/4 + 2	VUS D—potential interest CNV
15	Genome	~10% of aut. genome is IBD	Fetus with occipital encephalocele and postaxial polydactyly	NA	E/3 + 2	E—potential interest ROH
16	Genome	~10% of aut. genome is IBD	Fetus with normal ultrasound findings	NA	F/3 + 0	F—normal finding

*Clinical information column: A* = good match between known gene function and clinical phenotype, *B* = poor or no match between known gene function and clinical phenotype, *NA* not applicable, *B* benign, *LB* likely benign, *VUS* variant of unknown significance, *LP* likely pathogenic, *P* pathogenic, *IBD* identity by descent, aut. autosomal.

^a^Based on Bayesian score in Tavtigian et al. [25] for SNPs, and Riggs et al. [18] for CNVs (cnvcalc.clinicalgenome.org/cnvcalc/).

All cases are based on cases from routine diagnostic practice. The ACMG scores are derived from the genetic variant interpretation tool found on the web page of the University of Maryland School of Medicine [24]. This tool uses the Bayesian calculation developed by Tavtigian et al. [25] to reach an ACMG class (B, LB, VUS, LP, and P), and the ACMG scoring criteria included can also be found in Table 5. The ABC scores are based on data from selected cases from Suppl. Table S1.

In cases 1–6, the findings were heterozygosity for a single variant in a gene in a likely dominant clinical setting. In all cases, the results are in the same range, e.g., an ACMG-VUS or LB corresponding to ABC classes D or E. One difference is that the ABC system can be linked to standard comments answering a clinical question. Note that the *F5* Leiden mutation (case 1), which is not a VUS, ends up as an ACMG-VUS if you just use the Bayesian calculation system uncritically (which, of course, you should not do). The problem using the BA1 criterium (an MAF > 0.05 stand-alone variant is likely benign) has been addressed by a ClinGen working group, and a list of at least nine exemptions were made [14]. Only in case 6, there is a clinically important difference between ACMG (LP) and ABC system (E) grading. The reason is that “de novo” and “unique variant” counts more heavily in the ACMG system than a genotype–phenotype match. The same type of discrepancy, but with an opposite result, is found in case 9, where the good clinical match in the ABC system, classifying the variant as “pathogenic,” overrules the VUS score of the ACMG system.

In cases 7–11, the clinical question relates to a recessive disease with from one to three variants found in a known recessive gene. When only one variant was found in a possible recessive setting (cases 7, 10, and 11), the a priori likelihood that the tentative diagnosis is correct is very important [26]. In cases 7 and 11, it is likely that a second pathogenic variant in the other allele has not been found, unlike in case 10, where the *CFTR* mutation probably is a random finding. Although this does not affect the variant’s functional step A grading (Table 5), it affects the clinical step B grading and the choice of variant standard comment (step C; a category E or D choice from Table 4). When variants are found in both alleles (cases 8 and 9), each variant is graded separately, and the final laboratory report is then based on these scores and the clinical question (see further examples in Suppl. Table S1). For variant databases, the functional grading is the most invariable and should be reported.

Finally, Table 5 includes five cases (12–16), of which three also have been scored by the recently published and quite complicated CNV classification adaption to the ACMG system: cnvcalc.clinicalgenome.org/cnvcalc [18]. The first three are CNVs, one unique (case 12) and two recurrent (cases 13 and 14), and the last two exemplify the finding of extensive identity by descent (IBD), seen as several large homozygous stretches (ROHs) on multiple chromosomes after SNP-based array testing. Because the ABC system is linked to the clinical question and the phenotype, it is easy to score CNVs, even when quite benign and possibly randomly associated (like in case 14). For the high-degree IBD cases, the a priori likelihood that the phenotype has a recessive cause determines the step B grading and comment (step C), but not the step A grading, similar to some of the recessive cases (cases 7, 10, and 11). It is also unproblematic to score carrier status of an LoF variant in an imprinted gene, like shown for the *UBE3A* gene in Suppl. Table S1. This table also gives further examples on ABC variant classification, each class with alternative standard comments.

## Discussion

A good system for variant classification is essential for mainstreaming precision medicine. Such a system must continuously be improved, based on experience and user feedback. The prevailing system is developed by ACMG and AMP. It is one-dimensional with five classes, from benign to pathogenic, and classification is based on a mixture of molecular, functional, statistical, and clinical data. If the criteria for a class are not listed, it is not easy to know what, e.g., a VUS classification is based on. Furthermore, the VUS group is a mixture of variants with insufficient knowledge to allow classification (true unknowns), variants with conflicting evidence, and variants that do not reach a 90% likelihood for pathogenicity (sometimes called the “VUS+” or “hot VUS” group). Penetrance is not a criterium, but the classification is easier if the variant is dominant and highly penetrant. The system is not well suited for classifying complex dominant conditions, recessive diseases or hypomorphic alleles, and it was not designed for cytogenetics [16].

**Fig. 2:**
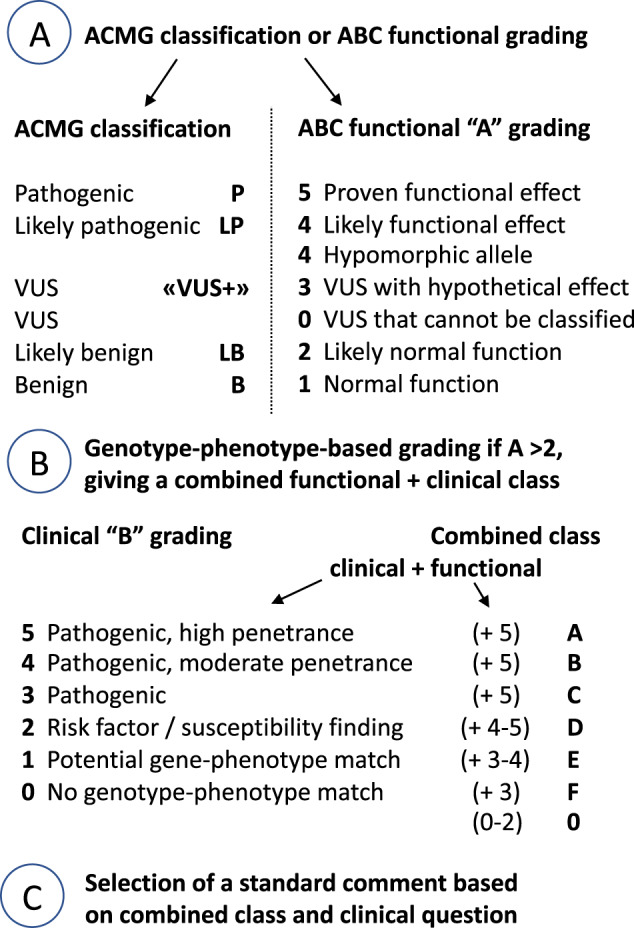
The ABC system can be used independently or as an add-on to the ACMG-based classification system. In the latter case, the VUS class is given a 0 (fVUS) or 3 (HFE) grade in step A of the ABC system, other classes are kept (P = 5, LP = 4, LB = 2, and B = 1). In the second step B of the ABC system, the issue is really how well the genotype and phenotype match, from 0 (no conceivable correlation) to 5 (perfect match). The combined class is given by the steps A and B grading and divided into seven classes: 0 if no step B grading was deemed necessary, and A–F if step B has been done after step A. In step C (optional), each class can be linked to the standard comments that best addresses the clinical question.

The ACMG system has been extended from 33 to 108 criteria by Nykamp et al. [23] and they call their system Sherloc. Sherloc appears to have many classification principles in common with the ABC system, like separate classification of functional and clinical data, with more weight given to the latter. However, what Sherloc calls clinical data are generally considered in step A functional grading of the ABC system. Therefore, both ACMG and Sherloc classification could easily be done first, followed by the stepwise ABC classification suggested here for the VUS group or other challenging variants (Fig. 2). The clinical step 2 part of the ABC system takes the gene-to-phenotype match into consideration, which is especially important when evaluating the result of broad gene panel testing.

Because the ACMG criteria can be weighted, a Bayesian calculator tool has been developed to aid class determination [25]. The use of this calculator is convenient and introduces a feeling of objectivity, but the result could be misleading. Usually, however, the result is as expected, and Bayesian-calculated ACMG classes and ABC classes mostly overlap (Table 5). One should, nevertheless, be aware that the calculator has fixed the a priori likelihood for a variant in a gene being causative at 0.10, which is too low if there is reason to suspect a specific gene (like *CFTR* in nasal polyposis, see Table 5) and too high if a large gene panel is examined (like genes associated with developmental delay). Figure 1 in their article nicely illustrates how the Bayesian calculations are prior probability dependent [25]. In addition, the odds of pathogenicity (from very strong to supportive) linked to the various ACMG criteria is the square root of the one above, starting with very strong that is given an odds of pathogenicity of 350, to strong at 18.7, to moderate at 4.3, and finally to supportive at 2.08 [20]. Naturally, this relationship does not reflect a biological reality but is an approximation that fits with conceived biological and clinical probability in many cases. Because of these caveats, and because gene panels tend to get larger (many contain from 500 to 2500 genes), we do not want to link the ABC system to a Bayesian calculator that is based on too many approximations and maybe false assumptions. We also think that the ABC classification system described here makes the use of such semi-objective tools less necessary.

The ABC system aims to make classification clearer by separating the functional consequence the variant has on the gene product from the phenotypic consequence the variant has for the individual (Fig. 2). The gene-to-phenotype match is the essence of the clinical classification (step B), and the final result can be linked to standard comments (step C, examples are found in Table 4). A further advantage is that all types of genetic variation can be classified, including CNVs, extensive homozygosity, regulatory changes, and hypomorphic alleles. If the condition in question could be recessive, each variant is scored separately, and the clinical report then takes the number of variants found and the prior probability for recessive disease into consideration (Table 5 and Suppl. Table S1). It is also possible to score “protective variants” during functional grading, e.g., if an allele diminishes the likelihood for a given condition. This category could be added to Table 1 and graded minus 1.

We find it useful that each ABC class (from A to F) is linked to from one to four standard comments, depending on the clinical question (Fig. 2 and Table 4). Importantly, these comments are flexible and can be designed by the specific user to best serve their practice and policy. They are not a compulsory part of the ABC classification system, just an add-on, i.e., a step C. The appropriate use of the ABC system usually requires close collaboration between the clinical laboratory geneticist (doing the functional grading) and the clinical geneticist (doing the clinical grading) or a medical specialist with expertise on the condition in question and its genetic causes. For this to work, clinical geneticists or collaborating specialists must have adequate molecular knowledge, otherwise, genotype–phenotype questions will be too poorly understood.

Although the ACMG and ABC system classes overlap in most cases where both systems can be used (Table 5), the ABC system has the advantage that the VUS group is split into a true VUS group (usually not reported) and VUS+ group (could be reported as a VOI, see Table 4). In a few cases, the systems disagree like in case 6, where the clinical picture is not likely to be caused by a de novo *CACNA1C* C-terminal tail variant, and in case 9, where the clinical diagnosis is Fowler syndrome, a quite unique recessive clinical condition. The ABC system is more helpful for the clinician also in these cases. We believe that our proposed system will be more informative also for non-expert primary care physicians and ultimately help to establish genomic medicine into the routine clinical care.

The system has been tested at the Department of Medical Genetics at Haukeland University Hospital (Suppl. Table S1) and also in a recent publication on variants associated with fetal akinesia [27]. A prototype of the system has been on the ESHG website (www.eshg.org) since July 2019 to obtain feedback from others, and useful feedback has also been obtained from the ESHG Board. It is so far not an ESHG Board-endorsed classification system. Hopefully, this new system, which can be independent or complementary to ACMG/Sherloc-based classification, will make gene-, disease-, or variant-type-dedicated systems less necessary and thus facilitate the mainstreaming of precision medicine.

## Supplementary information

Suppl Figure 1

Suppl Table S1

## References

[1] Amendola LM, Jarvik GP, Leo MC, McLaughlin HM, Akkari Y, Amaral MD (2016). Performance of ACMG-AMP Variant-Interpretation Guidelines among nine laboratories in the Clinical Sequencing Exploratory Research Consortium. Am J Hum Genet.

[2] Kanavy DM, McNulty SM, Jairath MK, Brnich SE, Bizon C, Powell BC (2019). Comparative analysis of functional assay evidence use by ClinGen Variant Curation Expert Panels. Genome Med.

[3] Vail PJ, Morris B, van Kan A, Burdett BC, Moyes K, Theisen A (2015). Comparison of locus-specific databases for BRCA1 and BRCA2 variants reveals disparity in variant classification within and among databases. J Community Genet.

[4] Harrison SM, Dolinsky JS, Knight Johnson AE, Pesaran T, Azzariti DR, Bale S (2017). Clinical laboratories collaborate to resolve differences in variant interpretations submitted to ClinVar. Genet Med.

[5] Yang S, Lincoln SE, Kobayashi Y, Nykamp K, Nussbaum RL, Topper S (2017). Sources of discordance among germ-line variant classifications in ClinVar. Genet Med.

[6] Fortuno C, Pesaran T, Dolinsky J, Yussuf A, McGoldrick K, Goldgar D et al. Differences in patient ascertainment affect the use of gene-specified ACMG/AMP phenotype-related variant classification criteria: evidence for TP53. Hum Mutat. 2020;41:1555–62. 10.1002/humu.23972 31898864

[7] Kelly MA, Caleshu C, Morales A, Buchan J, Wolf Z, Harrison SM (2018). Adaptation and validation of the ACMG/AMP variant classification framework for MYH7-associated inherited cardiomyopathies: recommendations by ClinGen’s Inherited Cardiomyopathy Expert Panel. Genet Med.

[8] Lee K, Krempely K, Roberts ME, Anderson MJ, Carneiro F, Chao E (2018). Specifications of the ACMG/AMP variant curation guidelines for the analysis of germline CDH1 sequence variants. Hum Mutat.

[9] Mester JL, Ghosh R, Pesaran T, Huether R, Karam R, Hruska KS (2018). Gene-specific criteria for PTEN variant curation: recommendations from the ClinGen PTEN Expert Panel. Hum Mutat.

[10] Nicora G, Limongelli I, Gambelli P, Memmi M, Malovini A, Mazzanti A (2018). CardioVAI: an automatic implementation of ACMG-AMP variant interpretation guidelines in the diagnosis of cardiovascular diseases. Hum Mutat.

[11] Oza AM, DiStefano MT, Hemphill SE, Cushman BJ, Grant AR, Siegert RK (2018). Expert specification of the ACMG/AMP variant interpretation guidelines for genetic hearing loss. Hum Mutat.

[12] Parsons MT, Tudini E, Li H, Hahnen E, Wappenschmidt B, Feliubadalo L (2019). Large scale multifactorial likelihood quantitative analysis of BRCA1 and BRCA2 variants: an ENIGMA resource to support clinical variant classification. Hum Mutat.

[13] Harrison SM, Biesecker LG, Rehm HL (2019). Overview of specifications to the ACMG/AMP Variant Interpretation Guidelines. Curr Protoc Hum Genet.

[14] Ghosh R, Harrison SM, Rehm HL, Plon SE, Biesecker LG (2018). ClinGen Sequence Variant Interpretation Working G: updated recommendation for the benign stand-alone ACMG/AMP criterion. Hum Mutat.

[15] Richards S, Aziz N, Bale S, Bick D, Das S, Gastier-Foster J (2015). Standards and guidelines for the interpretation of sequence variants: a joint consensus recommendation of the American College of Medical Genetics and Genomics and the Association for Molecular Pathology. Genet Med.

[16] Najafi A, Caspar SM, Meienberg J, Rohrbach M, Steinmann B, Matyas G (2020). Variant filtering, digenic variants, and other challenges in clinical sequencing: a lesson from fibrillinopathies. Clin Genet.

[17] Brandt T, Sack LM, Arjona D, Tan D, Mei H, Cui H, et al. Adapting ACMG/AMP sequence variant classification guidelines for single-gene copy number variants. Genet Med. 2020;22:336–44. 10.1038/s41436-019-0655-2 31534211

[18] Riggs ER, Andersen EF, Cherry AM, Kantarci S, Kearney H, Patel A (2020). Technical standards for the interpretation and reporting of constitutional copy-number variants: a joint consensus recommendation of the American College of Medical Genetics and Genomics (ACMG) and the Clinical Genome Resource (ClinGen). Genet Med.

[19] Maxwell KN, Hart SN, Vijai J, Schrader KA, Slavin TP, Thomas T (2016). Evaluation of ACMG-Guideline-Based Variant Classification of cancer susceptibility and non-cancer-associated genes in families affected by breast cancer. Am J Hum Genet.

[20] Tsai GJ, Ranola JMO, Smith C, Garrett LT, Bergquist T, Casadei S (2019). Outcomes of 92 patient-driven family studies for reclassification of variants of uncertain significance. Genet Med.

[21] Fokkema I, van der Velde KJ, Slofstra MK, Ruivenkamp CAL, Vogel MJ, Pfundt R (2019). Dutch genome diagnostic laboratories accelerated and improved variant interpretation and increased accuracy by sharing data. Hum Mutat.

[22] Pollard S, Sun S, Regier DA (2019). Balancing uncertainty with patient autonomy in precision medicine. Nat Rev Genet.

[23] Nykamp K, Anderson M, Powers M, Garcia J, Herrera B, Ho YY (2017). Sherloc: a comprehensive refinement of the ACMG-AMP variant classification criteria. Genet Med.

[24] Kleinberger J, Maloney KA, Pollin TI, Jeng LJ (2016). An openly available online tool for implementing the ACMG/AMP standards and guidelines for the interpretation of sequence variants. Genet Med.

[25] Tavtigian SV, Greenblatt MS, Harrison SM, Nussbaum RL, Prabhu SA, Boucher KM (2018). Modeling the ACMG/AMP variant classification guidelines as a Bayesian classification framework. Genet Med.

[26] Biesecker LG (2019). Genomic screening and genomic diagnostic testing-two very different kettles of fish. Genome Med.

[27] Pergande M, Motameny S, Ozdemir O, Kreutzer M, Wang H, Daimaguler HS et al. The genomic and clinical landscape of fetal akinesia. Genet Med. 2020;22:511–23. 10.1038/s41436-019-0680-1 31680123

