# Supplementary figures and images for: Stepwise ABC system for classification of any type of genetic variant
Source: Eur J Hum Genet. 2021 May 13;30(2):150–9. doi: 10.1038/s41431-021-00903-z (PMC8821602; doi:10.1038/s41431-021-00903-z)

# Functional grading

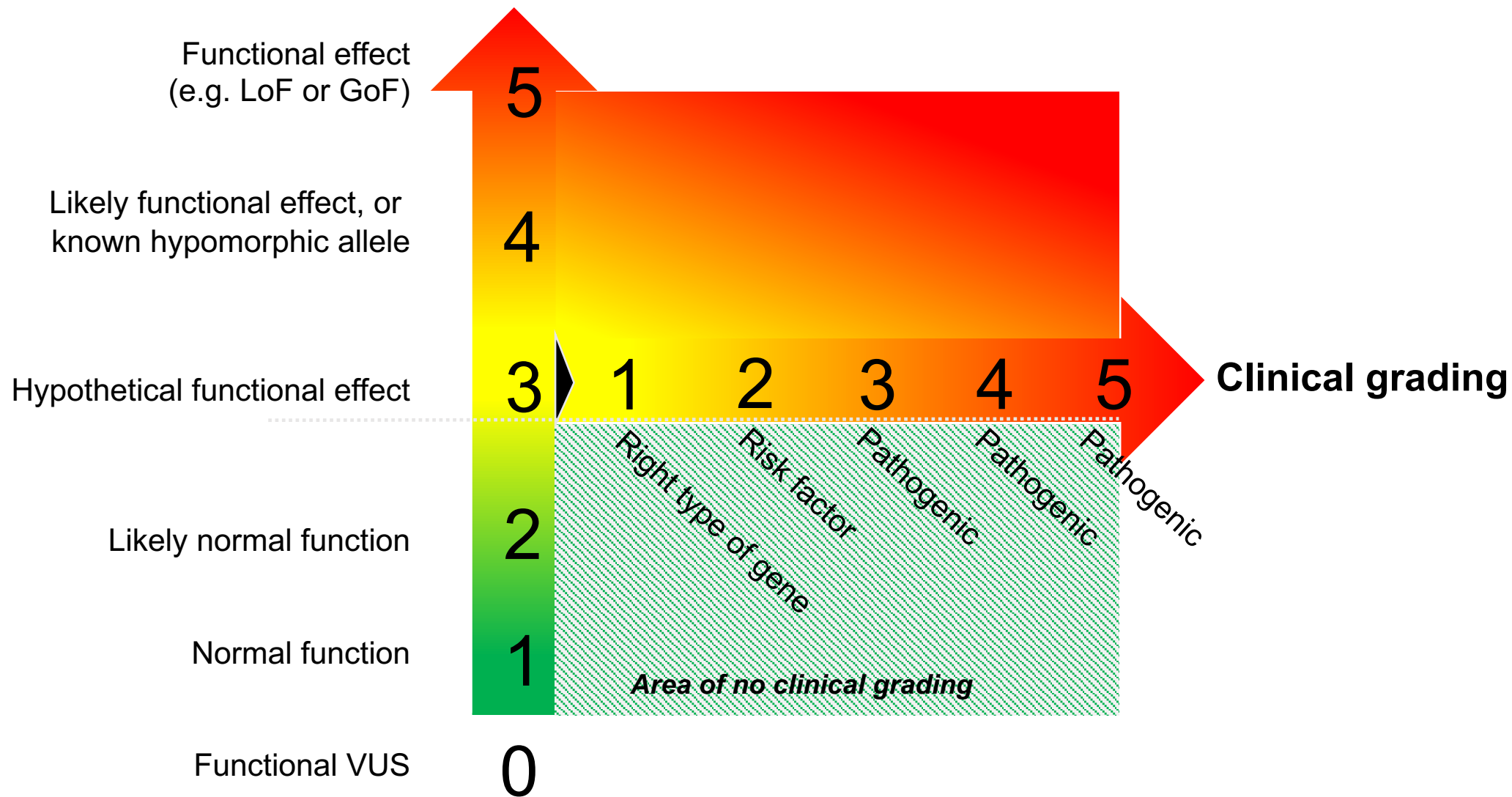

Supplement: Supplementary file 1 — Suppl Figure 1 [file 41431_2021_903_MOESM1_ESM.pdf]
